# Distribution and genomic characterization of tigecycline-resistant *tet*(X4)-positive *Escherichia coli* of swine farm origin

**DOI:** 10.1099/mgen.0.000667

**Published:** 2021-10-25

**Authors:** Yan Li, Qian Wang, Kai Peng, Yuan Liu, Xia Xiao, Mashkoor Mohsin, Ruichao Li, Zhiqiang Wang

**Affiliations:** ^1^​ Jiangsu Co-Innovation Center for Prevention and Control of Important Animal Infectious Diseases and Zoonoses, College of Veterinary Medicine, Yangzhou University, Yangzhou, Jiangsu Province, PR China; ^2^​ Institute of Comparative Medicine, Yangzhou University, Yangzhou, Jiangsu Province, PR China; ^3^​ Institute of Microbiology, University of Agriculture, Faisalabad, Pakistan

**Keywords:** *Escherichia coli*, genetic environment, nanopore sequencing, plasmid, *tet*(X4)

## Abstract

Abstract

The emergence of plasmid-mediated tigecycline-resistant strains is posing a serious threat to food safety and human health, which has attracted worldwide attention. The tigecycline resistance gene *tet*(X4) has been found in diverse sources, but the distribution of *tet*(X4) and its genetic background in the animal farming environment is not fully understood. Thirty-two *tet*(X)-positive *Escherichia coli* strains isolated from 159 samples collected from swine farms showed resistance to tigecycline. The *tet*(X)-positive strains were characterized by antimicrobial susceptibility testing, conjugation assay, PCR, Illumina and long-read Nanopore sequencing, and bioinformatics analysis. A total of 11 different sequence types (STs) were identified and most of them belonged to phylogroup A, except ST641. In total, 196 possible prophage sequences were identified and some of the prophage regions were found to carry resistance genes, including *tet*(X4). Furthermore, our results showed possible correlations between CRISPR spacer sequences and serotypes or STs. The co-existence of tigecycline-resistant *tet*(A) variants and *tet*(X4) complicates the evolution of vital resistance genes in farming environments. Further, four reorganization plasmids carrying *tet*(X4) were observed, and the formation mechanism mainly involved homologous recombination. These findings contribute significantly to a better understanding of the diversity and complexity of *tet*(X4)-bearing plasmids, an emerging novel public health concern.

## Data Summary

All data can be found under BioProject PRJNA663118 and PRJNA576562. The individually analysed Nanopore data-only plasmid sequences were deposited in the figshare database (https://doi.org/10.6084/m9.figshare.16843717) for reference.

**Impact Statement**

In this study, we described and characterized strains harbouring *tet*(X4) collected from environmental samples of pig farm origin through different techniques. Multiple resistance genes and virulence factors were detected on prophage and the *tet*(X4) gene was identified in the prophage region for the first time in the present study. Notably, we identified a novel *tet*(A) variant, which could decrease the effectiveness of tigecycline. It is interesting to note that plasmids carrying the *tet*(X4) gene with the same plasmid replicon type were isolated from samples of different sources in the pig farm environment, which indicates that plasmids carrying *tet*(X4) could transfer between different hosts. This study warned us to increase monitoring of the transmission of emerging novel resistance genes from animals to humans.

## Introduction

Antibiotic resistance poses a serious threat to human health and food security [1]. There are increasing reports suggesting that various environments could serve as important reservoirs of antibiotic resistance genes (ARGs), so ARGs have been recognized as novel environmental hazards [2, 3], and resistance genes can spread widely via water, soil and animal faeces [4, 5]. Tigecycline is considered to be a last-resort antibiotic for treating serious infections caused by multi-drug resistant (MDR) Gram-negative and Gram-positive bacteria [6, 7]. Tigecycline resistance has occurred sporadically in the past few years, primarily due to the overexpression of non-specific active efflux pumps and ribosome protection [8]. Recently, a novel plasmid-mediated tigecycline resistance mechanism, conferred by Tet(X3) and Tet(X4), has been described in *Enterobacterales* and *Acinetobacter* isolates from different sources in China [7, 9, 10], which indicates that *tet*(X) located on mobile elements is becoming a serious threat. However, the genomic epidemiology of environmental *tet*(X)-bearing bacteria has been largely unknown, especially in veterinary settings.

With great advancements in DNA sequencing technologies and decreasing sequencing costs accordingly, next-generation sequencing (NGS) has been widely used in bacterial genomic investigations [11]. However, the *tet*(X4) gene is usually located in complex structures that are difficult to obtain as complete maps depending solely on short-read NGS methods [12]. Understanding the complex structure and transmission routes of the *tet*(X) gene is critical for comprehending molecular epidemiology and evolution among different sectors as a One Health approach. Nanopore sequencing, mainly due to its long read length and easy availability, is being used extensively in analysing complex multi-drug resistance structures [13–15]. Accordingly, we employed Nanopore long-read sequencing to characterize and trace the *tet*(X)-positive strains from a pig farm in China.

Bacteriophages (phages) are viruses of bacteria that specially infect and subsequently lyse the host bacteria [16]. Based on their reproduction, the phages are usually divided into lytic phages and temperate phages [17]. These two phages play important roles in bacterial evolution or diversification by horizontal gene transfer [18]. Existing data suggest that phages make a significant contribution to the dissemination of resistance genes [19]. Further, intact or remnant prophage elements have been identified in bacterial genomes and can even account for up to 20% of the bacterial genome in some strains [20]. Hence, the identification of phages in bacterial genomes could help us to understand horizontal gene transfer of resistance genes, especially emerging novel resistance genes.

The increasing number of resistance genes discovered in the environment has become an important ecological issue [21, 22]. PR China is one of the largest pig-rearing nations in the world, and in-depth investigation of the *tet*(X) gene in pig farm environments is vital to determine the fundamental dissemination of these genes and set up a reasonable control framework. Here, we analyse the *tet*(X)-positive *Escherichia coli* isolated from a pig farm in Jiangsu province, PR China in 2018, illustrate multiple intricate genetic environments of *tet*(X4) and show that the pig farming environment is an important repository of hazardous *tet*(X4).

## Methods

### Bacterial isolates

A total of 159 samples including swine faeces (*n*=36), swine anus (*n*=36), swine nose (*n*=36), wastewater (*n*=30) and soil (*n*=21) were collected from a pig farm in Jiangsu province, PR China in 2018. The samples were stored in ice boxes during transfer to our laboratory for further processing. The samples were cultured in 5 ml Luria–Bertani (LB) broth containing tigecycline (4 mg l^−1^) and incubated at 37 °C for 6 h in a shaking incubator to enrich tigecycline-resistant microbiota. The enriched cultures were streaked onto MacConkey agar plates containing tigecycline (4 mg l^−1^) to screen the tigecycline-resistant isolates. The selected single colonies were further purified and stored in LB broth containing 20% glycerol at −80 °C. The positive isolates were subjected to 16S rRNA sequencing for species identification. The primers used were 16 S-F: AGAGTTTGATCATGGCTC; 16 S-R: GGTTACCTTGTTACGACTT. The *tet*(X) resistance genes were determined by PCR with reported primers [7].

### Antimicrobial susceptibility testing (AST)

AST was performed using the broth microdilution method, and *E. coli* ATCC 25922 was used as the quality control (Table S1). The MICs of tigecycline and other antimicrobials were determined and interpreted using Clinical and Laboratory Standards Institute (CLSI) guidelines and the resistance breakpoints were interpreted according to the European Committee on Antimicrobial Susceptibility Testing (EUCAST) criteria (>2 mg l^−1^) for tigecycline and CLSI guidelines [23] for the remaining antibiotics.

### Conjugation experiments

To investigate the transferability of *tet*(X4), a conjugation assay was conducted using rifampicin-resistant *E. coli* EC600 or sodium azide-resistant *E. coli* J53 as recipients. Donor and recipient broth cultures were mixed in a ratio of 1:4, and cultivated on LB agar plates at 37 °C overnight. Transconjugants were selected on LB agar plates containing double antibiotics (tigecycline 4 mg l^−1^, rifampicin 300 mg l^−1^ or tigecycline 4 mg l^−1^, sodium azide 200 mg l^−1^) and confirmed with PCR. The plasmid profiles were characterized by S1-PFGE [24].

### Whole genome sequencing

The genomes of tigecycline-resistant strains were extracted with the TIANamp Genomic DNA kit (TianGen, Beijing, PR China) according to the manufacturer’s recommendations and quantified by the Qubit 4 Fluorometer. Genomic DNA (OD260/280≈1.8) was sequenced using the Illumina Hiseq 4000 platform with 150 bp paired-end sequencing. The plasmids in transconjugants were extracted with the Qiagen Plasmid Midi kit, following the manufacturer’s instructions. Subsequently, according to the results of MICs, Illumina sequencing data and S1-PFGE, representative strains were selected and subjected to third-generation long-read Nanopore sequencing. Libraries were constructed using the rapid barcoding kit RBK004 and subjected to ONT long-read sequencing in a MinION sequencer with the R9.4.1 flow cell according to the protocol (version RBK_9054_v2_revJ_14Aug2019) [25].

### Bioinformatics analysis

The paired-end short reads were *de novo*-assembled using SPAdes version 3.14.0 and the contigs <1 kb in length were removed using seqkit v.0.8.0. The genome sequences were completed with a hybrid *de novo* assembly strategy combining Illumina short-read and Nanopore MinION long-read data using Unicycler v.0.4.8 software [26]. For multi-drug resistance regions that could not be resolved by short-read data or even the hybrid assembly method, Nanopore long-read sequences were assembled by Flye v.2.4.2 software to acquire accurate structures of complex multi-drug resistance regions in genomes [27]. After obtaining draft or complete genomes, annotation was processed with the RAST pipeline using default parameters. The complete *tet*(X4) plasmids were characterized with Inc types. For the remaining isolates with only Illumina sequencing data, we mapped the draft genome assemblies against the reference plasmids obtained by Nanopore sequencing by using GView (https://server.gview.ca/) to infer their plasmid types and structures unequivocally.

Antimicrobial resistance genes were discovered using the online website ResFinder v.4.1 (https://cge.cbs.dtu.dk/services/ResFinder/) with default parameters. Plasmid replicons were detected using PlasmidFinder v.2.1 (https://cge.cbs.dtu.dk/services/PlasmidFinder/) with 95% minimum identity and 60% minimum coverage. Virulence genes were determined using the virulence factor database (last updated 14 October 2020) in ABRicate v.1.0.1 (https://github.com/tseemann/abricate) [28]. The serotypes of all *tet*(X)-positive *E. coli* were identified using SeroTypeFinder v.2.0.1 [29] and ECTyper v.1.0 software (https://github.com/phac-nml/ecoli_serotyping) with default parameters. Putative prophages in bacteria were predicted using the PHASTER tool [30]. Clustered regularly interspaced short palindromic repeats (CRISPRs) were searched using CRISPRFinder web database [31]. The core genome multi-locus sequence type (MLST) allelic profiles of *E. coli* were built using PHYLOViZ v.2.0 [32]. BRIG v.0.95 and Easyﬁg v.2.2.3 were used to generate plasmid comparison maps [33, 34].

In order to illustrate the evolutionary relatedness of strains in this study, phylogenetic trees of all *tet*(X4)-positive strains (*n*=32) collected from 29 samples were constructed using Parsnp v.1.2 based on the single-nucleotide polymorphisms (SNPs) of core genomes in the Harvest package with the 1000 bootstrap sampling value, and recombination filtering was conducted using PhiPack software [35]. Further, to clarify how 32 strains in this study fit within the *E. coli* lineage, a total of 88 *E. coli* genomes, 32 strains from this study and 56 strains from the National Center for Biotechnology Information (NCBI) and European Nucleotide Archive databases, were downloaded and plotted using Parsnp v.1.2. The resulting phylogeny was visualized and retouched using iTOL (https://itol.embl.de).

### Functional confirmation of *tet*(A)

To confirm the resistance function of the new *tet*(A) variant, TA cloning and AST were performed. The novel variant together with its promoter region were amplified by PCR using the primers TetA-v-F: CAGTCGTCGTCGGCTCTC and TetA-v-R: GATGCCTACAGGAACCAATG, cloned into pCE2 vector and transformed chemically into *E. coli* DH5α. Subsequently, the resistance phenotype of *tet*(A)-v to tigecycline was tested by broth microdilution and the resistance breakpoint was interpreted according to the EUCAST criteria (>2 mg l^−1^).

## Results and discussion

### Recognition and characterization of *tet*(X)-positive *Enterobacterales*

Among 159 samples, a total of 32 *tet*(X4)-positive strains out of 29 samples [3 samples generated 2 different *tet*(X4) clones] were obtained (18.24%) (Table S1), and no other *tet*(X) variants were identified. The positive rate for *tet*(X)-positive samples of this study was higher than that (6%) from other pig farms in Jiangsu Province [36]. The *tet*(X4) positive rate for strains was different in disparate sources. The anal swab (37.93%) and faeces (31.03%) had relatively high *tet*(X4)-positive rates, compared to other samples. Further, strains carrying *tet*(X4) had been also found in the environment of pig farms, such as water (6.89%) and soil (6.89%), which were possibly contaminated with *tet*(X4)-bearing intestinal microbiota. All of these 32 *tet*(X)-positive strains were subsequently identified as *E. coli* by 16S rRNA sequencing. Several papers have reported the discovery of *tet*(X4)-carrying *E. coli* [37–39], illustrating the fact that *E. coli* is an important carrier and indicator organism for the spread of *tet*(X4). Remarkably, two different strains carrying *tet*(X4) were isolated from each sample among three samples (Table S2), indicating that the *tet*(X4) gene could spread in the same microbiota. The MIC results demonstrated that these *tet*(X4)-positive strains were resistant to multiple tetracyclines, including tigecycline (Table S3). Further, they also were resistant to florfenicol and amoxicillin, and none of these isolates were resistant to meropenem or polymyxin. Moreover, all strains carrying *tet*(X4) showed a worryingly high level of resistance to tigecycline (8–64 mg l^−1^), which should attract much attention.

### Sequence types diversity of *tet*(X4)-positive *E. coli* strains

**Fig. 1.**
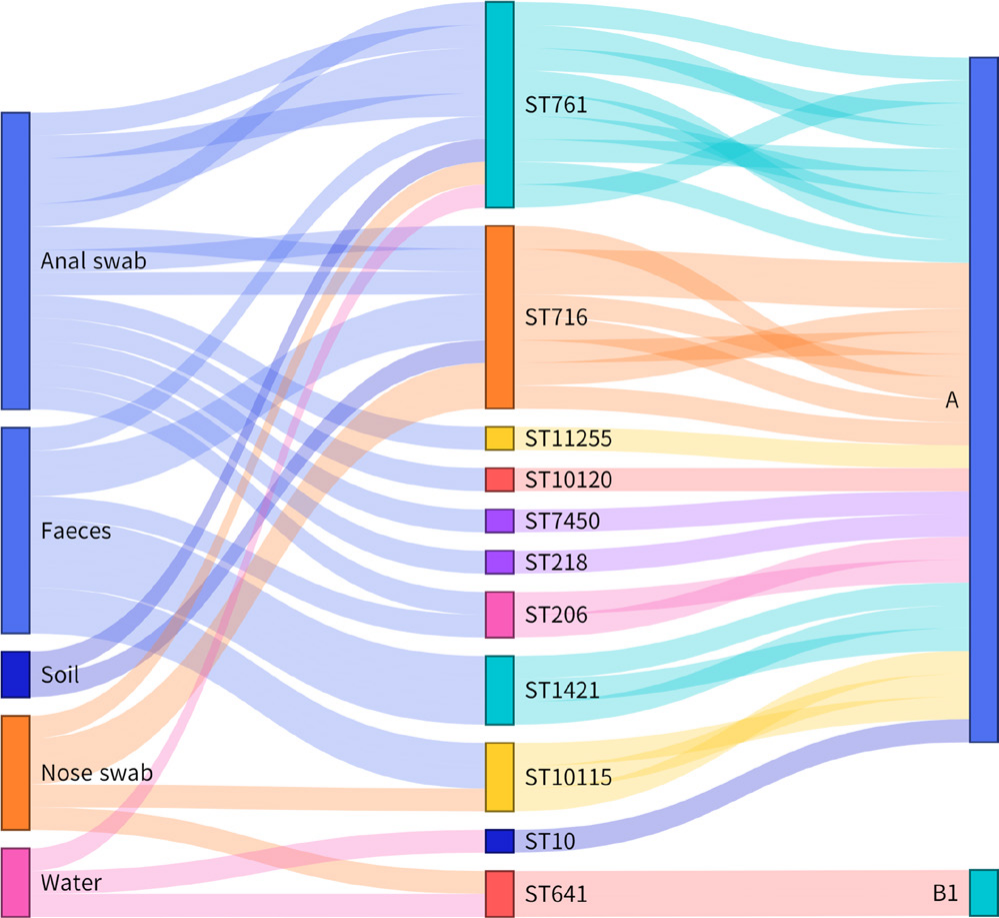
Sankey diagram demonstrating the *tet*(X4)-positive *E. coli* STs, phylogroups and the strain source. The lines are drawn connecting source, STs and phylogroups based on corresponding information from the 32 *tet*(X4)-positive strains. The diameter of the line is proportional to the number of isolates.

To better understand the constitution of these strains that carried *tet*(X4), whole genome sequencing was conducted for all identified *tet*(X4)-positive strains (Table S4). A total of 11 MLSTs with 3 new STs (ST10115, ST10120, ST11225) were acquired using *in silico* MLST analysis (Fig. 1). The prevalent STs in this study, ST761 (9/32, 28.13%) and ST716 (8/32, 25%), were different from those in previous reports [36]. Moreover, a high abundance of STs was found in the anal swab (7/11, 63.64%) and faeces (5/11, 45.45%). Various STs explained that *tet*(X4) is widespread in the pig farm environment.

### Phylogenetic analysis of *tet*(X4)-positive *E. coli*

**Fig. 2.**
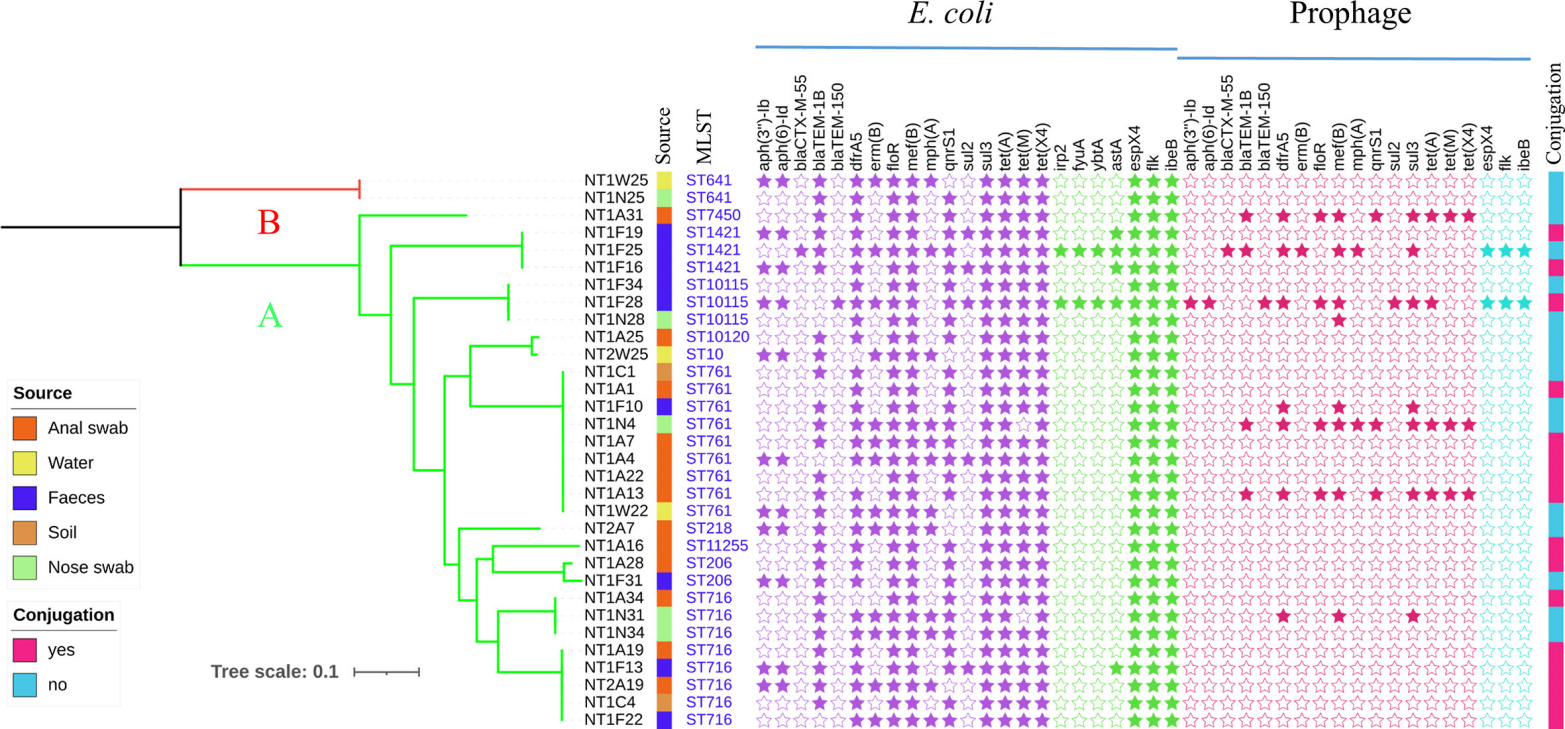
Core-genome phylogeny of 32 *tet*(X4)-positive *E. coli* strains. Maximum-likelihood analysis built from 94 029 non-recombinant core-genome single-nucleotide polymorphisms (SNPs) relative to the reference chromosome NT1F31 (GenBank: CP045190). SNPs were identified using Parsnp v1.2 with the PhiPack recombination filter and represent a 3 617 635 bp core-genome. Phylogeny is rooted according to the actual root by *Escherichia fergusonii* ATCC 35469 (GenBank: CU928158). The tree scale bar represents the number of nucleotide substitutions per site.

To further investigate the genetic relationship of *tet*(X4)-positive *E. coli *, a phylogenetic tree based on core-genome SNPs was constructed. According to Figs 2 and S1, only two phylogroups, A and B1, were detected in 32 *E. coli *. Our results indicated that A is the main phylogroup of *E. coli* isolated from pig farms, followed by phylogroup B1, consistent with previous research [40]. Further, reports indicated that commensal *E. coli* strains from group A predominated in the gut flora [40], which implied that *tet*(X4)-positive bacteria belonging to group A may spread to pig farm staff. The results for the phylogenetic tree and the MLST indicated that *tet*(X4)-positive *E. coli* from different sources in pig farms were various and there was no obvious clonal spread. Most of the strains on the same branch belonged to the same ST containing the same antibiotic resistance genes and virulence genes. In total, 24 different ARGs have been identified in tigecycline-resistant strains (Fig. S2) and all of them harboured the *floR*, *sul3* and *tet*(A) genes, which made the strains resistant to a variety of antibiotics and limited the therapeutic options. Further, these *tet*(X4)-positive strains contained diverse ARGs collected from different sources in pig farms, which further underlined the role of the environment in facilitating the dissemination of resistance genes.

In addition to resistance genes, these isolates harboured multiple crucial types of virulence factors. The high-pathogenicity island (HPI) of *Yersinia* was constituted by three genes, *fyuA*, *ybtA* and *irp2* genes, which were highly similar to *E. coli irp2* and *fyuA* genes [41]. *Yersinia* HPI was most prevalent in *E. coli* [42], but it was only observed in phylogroup B in this study, which implies that HPI has a lower prevalence in group A. Further, effector protein *espX1*, *espX4 espX5* and *espY1* genes, constituting the type III secretion system (T3SS), were also identified in *E. coli *. All 32 *E. coli* isolates carrying *tet*(X4) are resistant to at least 3 antimicrobials and possess a variety of virulence factors, which greatly increase the risk of co-transfer of multi-drug resistance and virulence genes. ARGs and virulence factors could transfer via mobile genetic elements such as plasmids, transposons and integrons, which further compounds the pollution problem of ARGs and other hazardous genetic materials in pig farms.

### Prophage analyses

A total of 196 probable prophage sequences were identified in all 32 *tet*(X4)-positive *E. coli *. According to the algorithm of the PHASTER tools, the prophage sequences can be divided into three categories, namely intact, incomplete and questionable prophages [30, 43]. As previously described, phage-mediated transfer of antibiotic resistance genes and virulence factors is known to be a vital mechanism for gene transfer between bacteria [44, 45]. In total, 82 intact prophages matched 39 known phage species, 95 incomplete prophages matched 25 known phage species, and 19 questionable prophages matched 10 known phage species (Fig. S3). In intact prophages, phages identified as Shigel_SfII-like were all detected in ST761 and ST1421 in the same lineages. Further, Salmon_SSU5-like were found in ST10115, ST206 and ST761 in the same branch. These observations clearly indicated that disparate prophage species of intact phages may relate to different *E. coli* lineages. In addition, the incomplete and questionable prophages are random distributions in these strains.

A total of 16 (*n*=16/24, 66.67%) different ARGs and diverse virulence factors were discovered in the prophage region (Fig. 2). This phenomenon illustrated that the prophage harbours numerous and diverse resistance genes, which should attract our attention. The 16 ARGs belonged to 10 different classes that include familiar clinical antimicrobial types, including aminoglycoside (*aph(3'')-Ib, aph(6)-Id*), beta-lactamase (*bla*_CTX-M-55_, *bla*_TEM-1_, *bla*_TEM-150_), trimethoprim (*drfA5*), macrolides‐lincosamide‐streptogramin [*erm*(B), *mef*(B)], chloramphenicol (*floR*), macrolide (*mphA*), fluoroquinolone (*qnrS1*), sulfonamide (*sul1, sul2, sul3*), tetracycline [*tet*(A), *tet*(M)] and tigecycline [(*tet*(X4)]. Furthermore, some virulence genes were also found in prophage genomes. Interestingly, these virulence factors were only observed in ST641 belonging to phylogroup B, which implies that virulence genes carried by prophages may be associated with STs or phylogroups.

### CRISPRs analysis of ***tet*** (X)-positive strains

The CRISPR arrays of 32 *tet*(X4)-positive strains were also identified and analysed. Direct repeats of 28–29 bp were separated by a diversified number (4–17) of spacer arrays. As previously reported, there are in general two subtypes of CRISPR systems in *E. coli *, I-E and I-F [46]. In this study, CAS type I-E subtypes were identified in 65.63% (21/32) of 32 strains genomes, which appears to match a previous study (Fig. S4) [46]. However, the remaining strains could not be classified because of missing cas genes. A total of 427 spacers were detected from genomes in 32 strains and 92 were unique spacers. Interestingly, 14 unique spacers from different plasmids or phage regions were discovered by using the blastn algorithm in GenBank (Table S5), which might provide immunity against these phages and plasmids. Currently, molecular typing methods based on CRISPR typing have been widely established in bacteria such as *Yersinia pestis* [47], *Campylobacter jejuni* [48] and *Salmonella* Enteritidis [49]. Further, several studies have reported that the spacers in CRISPR are related to serotyping and MLST typing [49–52]. Our results showed that the majority of *E. coli* in this study with the same serotype or ST had consistent spacer sequences. These results indicated that a certain correlation between the spacer sequences and ST or serotype in *E. coli* may exist.

### Amino acid sequence analysis of *tet*(A) gene variant

Notably, 32 *tet*(X4)-positive strains harbour the *tet*(A) gene and blast results indicated that the amino acid sequences of *tet*(A) [*tet*(A)-v and type 1] in our study are different from the first reported *tet*(A) gene (NCBI accession no. X00006). Previous studies have clearly shown that *tet*(A) variants play a significant role in tigecycline resistance strains [53, 54]. Apart from the type 1 *tet*(A) variant as previously reported to have been identified in one strain [53], we also identified a novel *tet*(A) variant designated *tet*(A)-v. The amino acid sequence of *tet*(A)-v has some similarity to that of the type 1 *tet*(A) variant, with only one amino acid difference (Fig. S5), which implies that the new *tet*(A) variant may also reduce the sensitivity of strains to tigecycline. According to the results of TA cloning and AST, the tigecycline MIC of *tet*(A)-negative carrier pCE2-DH5α was 0.25 µg ml^−1^, while the *tet*(A)-v-pCE2-DH5α was 1 µg ml^−1^, illustrating that the novel *tet*(A) variant could reduce the susceptibility of the strain to tigecycline. The genetic environment of *tet*(A)-v carried by plasmids was determined by blast. The *tet*(A)-v gene was located on 5 kb regions [△*tnpA*-relaxase-*tet*(R)-*tet*(A)-v-*pecM*-*hp*-△*tnpA*] and was found in all 32 *tet*(A)-positive strains, which showed high similarity to transposon Tn*1721* (Fig. S6). Further, 25 bp imperfect repeat regions with two mismatches were found on both sides of this sequence, illustrating that this genetic structure has the potential to transfer to other hosts and reduced susceptibility to tigecycline. The co-existence of tigecycline-resistant *tet*(A) variants and *tet*(X4) complicates the evolution of vital resistance genes in farming environments.

### Conjugation of ***tet*** (X4)-bearing genetic structures

Sixteen out of 32 strains carrying the *tet*(X4) gene were transferred successfully to the recipient bacterium, and all isolates from nose swabs and water were conjugated successfully, illustrating that the *tet*(X4) gene in these strains was located on conjugative genetic elements. S1-PFGE results showed that all transconjugants contain at least one plasmid, which illustrated that the *tet*(X4) gene was located on conjugative plasmids. Furthermore, the plasmids in four transconjugants were much larger than those in donor strains, suggesting that plasmid reorganizations may have occurred during the conjugative transfer.

**Fig. 3.**
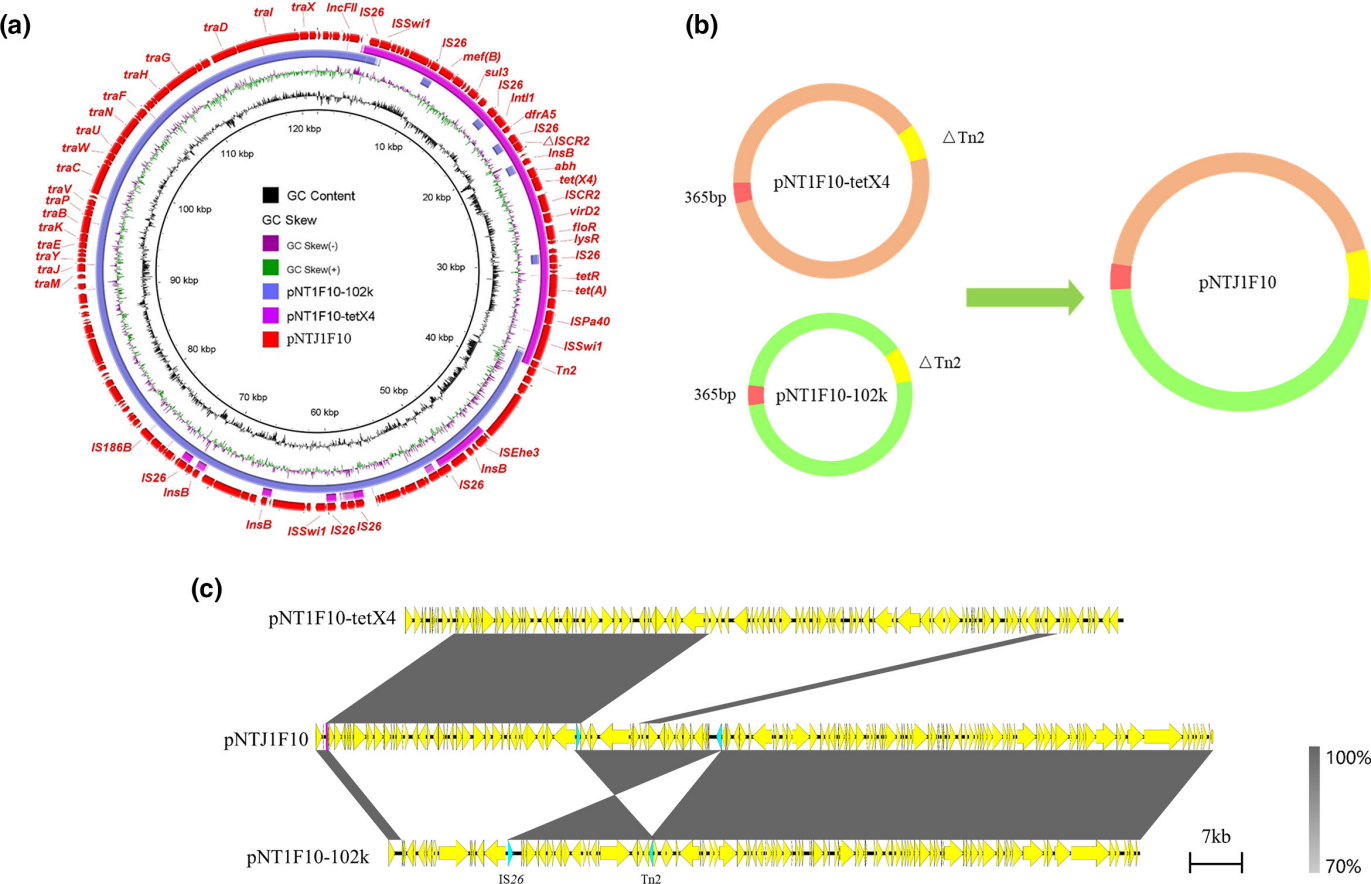
Fusion mechanism of plasmid pNTJ1F10. (**a**) Circular comparison between pNTJ1F10 in the transconjugant and its progenitor plasmids in the donor strain. The outmost circle denotes the reference plasmid pNTJ1F10 with annotated genes. (**b**) Schematic diagram of cointegrated plasmid generation mediated by homologous recombination. (**c**) Linear comparison between pNTJ1F10 and its progenitor plasmids. The grey regions indicate the homologous region between plasmid regions.

**Fig. 4.**
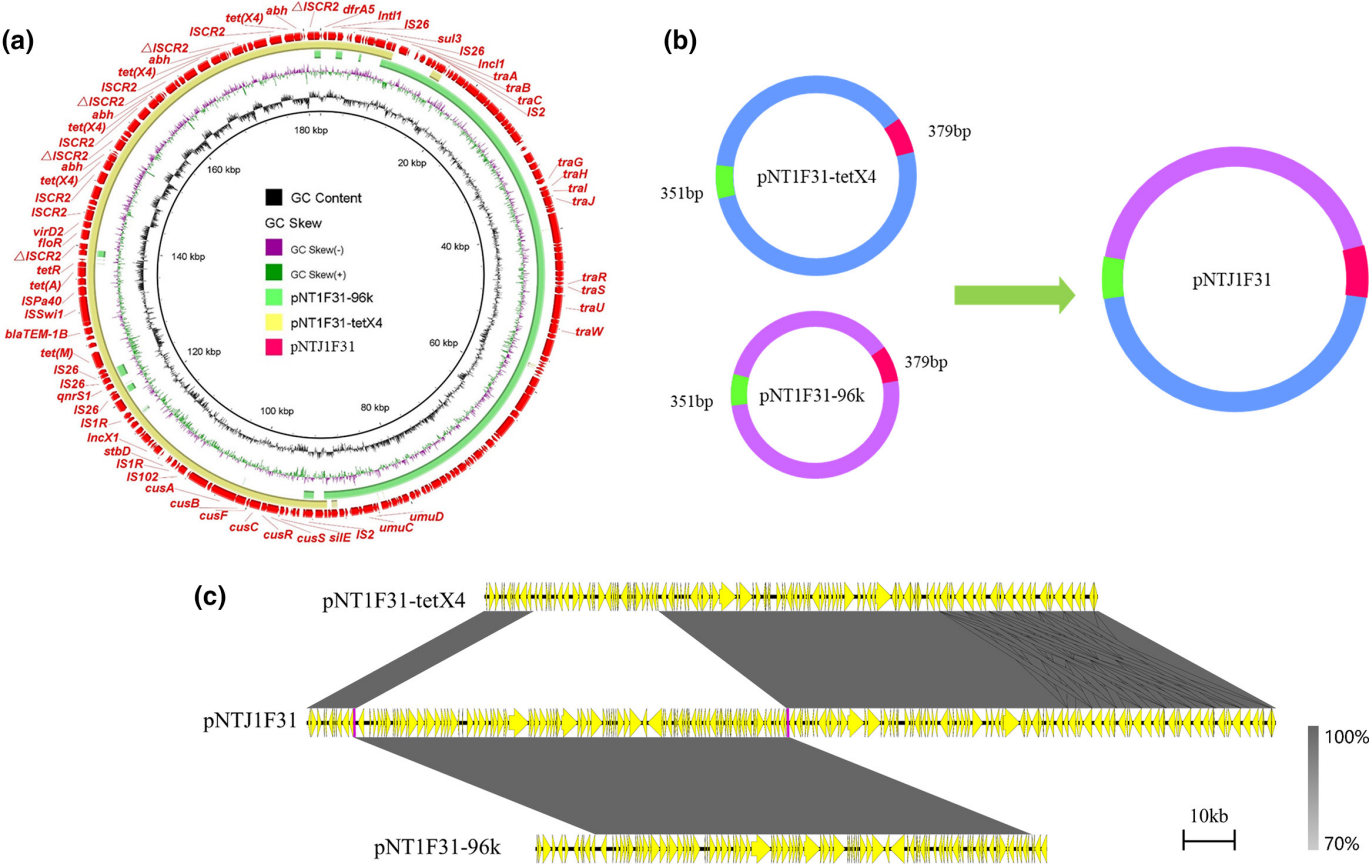
Fusion mechanism of plasmid pNTJ1F31. (**a**) Circular comparison between pNTJ1F31 in the transconjugant and its progenitor plasmids in the donor strain. The outmost circle denotes the reference plasmid pNTJ1F31 with annotated genes. (**b**) Schematic diagram of cointegrated plasmid generation mediated by homologous recombination. (**c**) Linear comparison between pNTJ1F31 and its progenitor plasmids. The grey regions indicate the homologous region between plasmid regions.

**Fig. 5.**
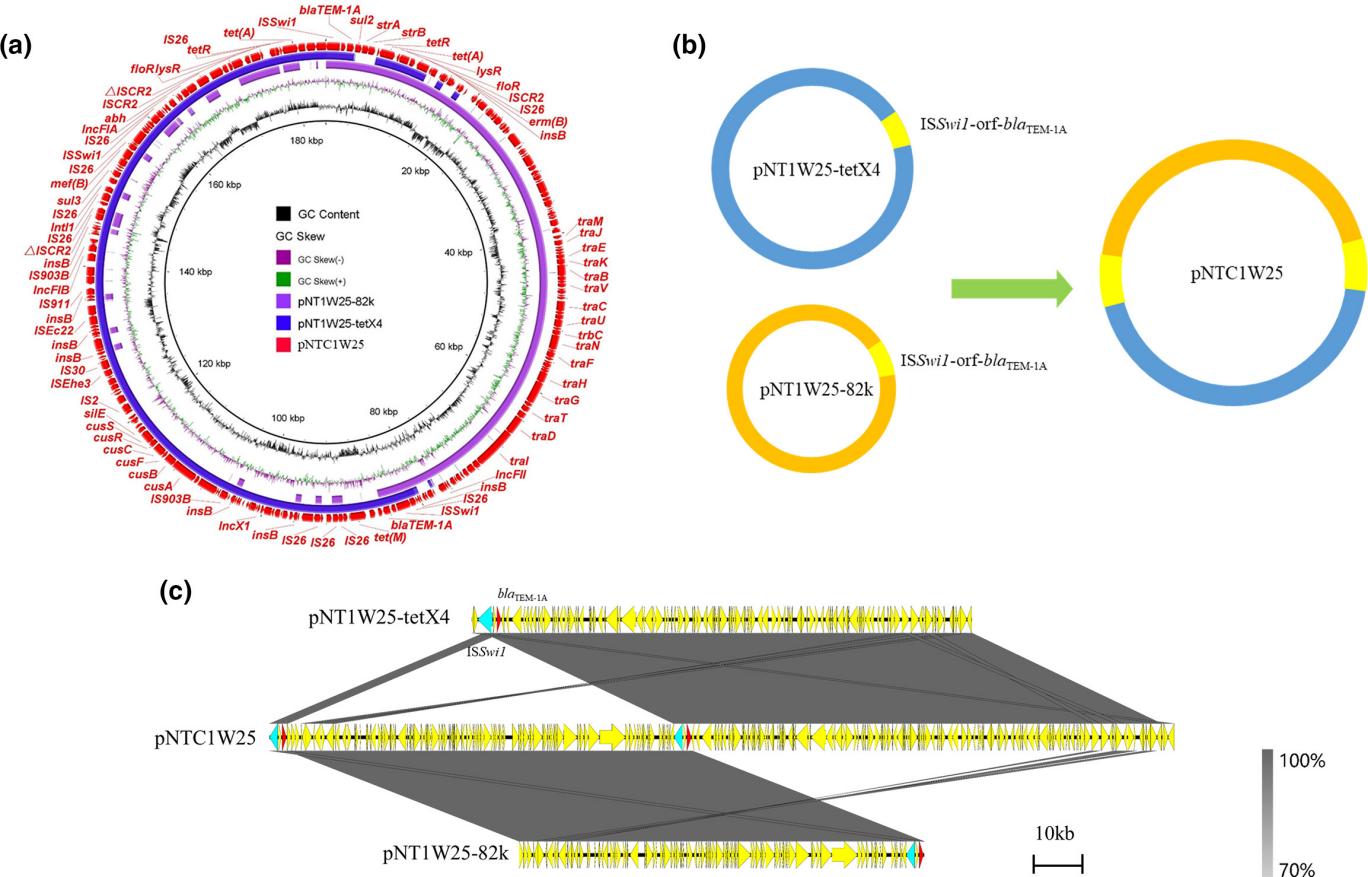
Fusion mechanism of plasmid pNTC1W25. (**a**) Circular comparison between pNTC1W25 in the transconjugant and its progenitor plasmids in the donor strain. The outmost circle denotes the reference plasmid pNTC1W25 with annotated genes. (**b**) Schematic diagram of cointegrated plasmid generation mediated by homologous recombination. (**c**) Linear comparison between pNTC1W25 and its progenitor plasmids. The grey regions indicate the homologous region between plasmid regions.

**Fig. 6.**
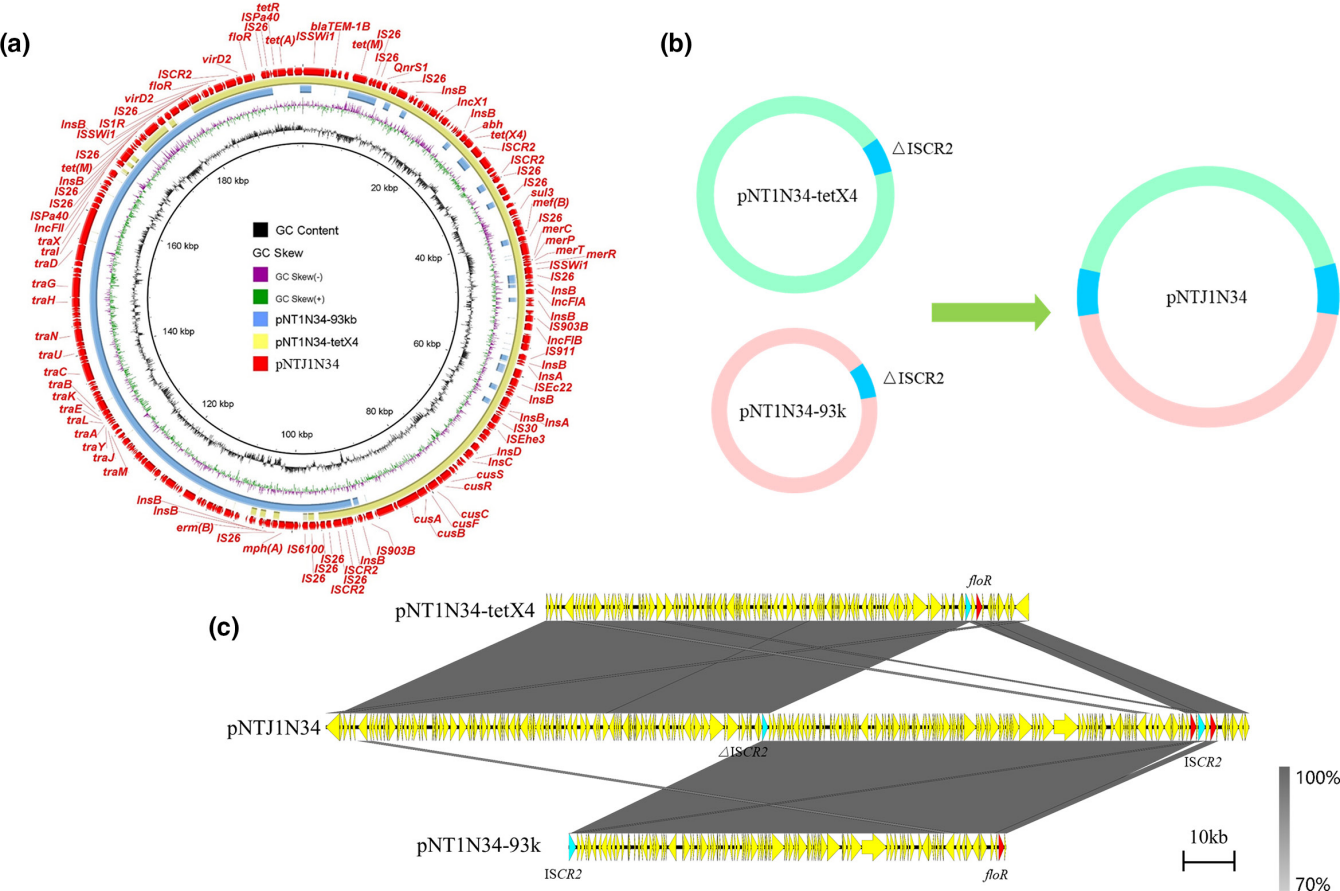
Fusion mechanism of plasmid pNTJ1N34. (**a**) Circular comparison between pNTJ1N34 in the transconjugant and its progenitor plasmids in the donor strain. The outmost circle denotes the reference plasmid pNTJ1N34 with annotated genes. (**b**) Schematic diagram of cointegrated plasmid generation mediated by homologous recombination. (**c**) Linear comparison between pNTJ1N34 and its progenitor plasmids. The grey regions indicate the homologous region between plasmid regions.

Plasmids of four transconjugants were sequenced using the MinION Nanopore long-read platform for further in-depth analysis. The formation of fusion plasmids is mainly mediated by homologous recombination and insertion sequences [24, 55]. The fusion mechanism of four transconjugants in this study was also formed by homologous recombination. The plasmid in transconjugant NTJ1F10 (Fig. 3) was generated by two plasmids pNT1F10-102k (IncFII) and pNT1F10-tetX4 (IncX1-IncFIA-IncFIB). Studies have reported the important role of IS*26* in mediating sequence rearrangement [56]. Compared with pNT1F10-102k, we found that the 20 kb region in pNTJ1F10 in the transconjugant NTJ1F10 terminated with IS*26* and Tn*2* reversed. To verify the correctness of this region, multiple long reads were confirmed to harbour the accurate 20 kb region (Fig. S7), implying that the sequence rearrangement may be mediated by mobile elements. In transconjugant NTJ1F31 (Fig. 4), pNTJ1F31 was generated by the fusion of pNT1F31–tetX4 (IncX1-IncFIA-IncFIB) and pNT1F31–96kb (IncI1) by homologous recombination. The plasmid pNTC1W25 in transconjugant NTC1W25 was formed by homologous recombination of pNT1W25–82k (IncFII) and pNT1W25–tetX4 (IncX1-IncFIA-IncFIB) through the shared genetic structure IS*Swi1*-orf-*bla*_TEM-1A_ (3638 bp) (Fig. 5). The generation of pNTJ1N34 (Fig. 6) was generated by plasmids pNT1N34–93k (IncFII) and pNT1N34–tetX4 (IncX1-IncFIA-IncFIB). The 2554 bp region of the *floR* gene in the two plasmids was integrated into the transconjugant, forming two *floR* gene tandem repeats carried by transconjugant. The *tet*(X4) gene is often found in multi-drug resistance regions [9, 25]; plasmids carrying *tet*(X4) could reorganize with other plasmids to form a fusion plasmid, which increases the risk of multi-drug resistance area dispersal.

### Genetic environment of plasmids harboring the *tet*(X4) gene

**Fig. 7.**
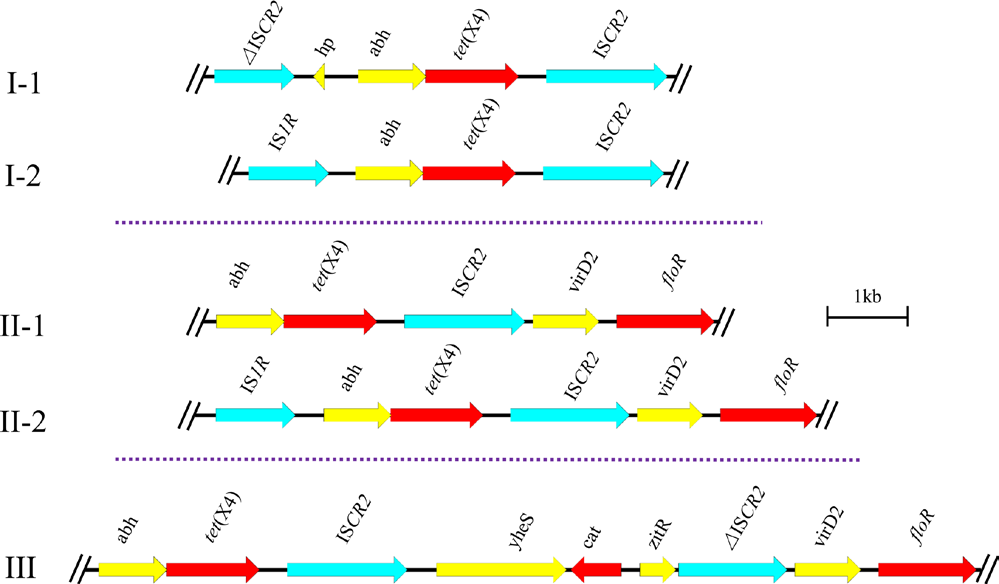
The different genetic environments of *tet*(X4). The red arrows and the blue arrows represent the resistance genes and insertion sequences, respectively. The arrows indicate the direction of transcription of the genes.

According to resistance phenotypes and WGS data, representative *tet*(X4)-positive strains were picked for third-generation Nanopore sequencing. The genetic contexts of *tet*(X4) were analysed and categorized into three main groups, suggesting that the surrounding genetic environments of *tet*(X4) are diverse and not limited by specific genetic backgrounds (Fig. 7). Group I (I-1, I-2) has the conserved structure *abh-tet*(X4)-IS*CR2*, which was classified in two types with different genes (IS*CR2,* IS*1R*) in the upstream region. Group II (II-1, II-2) has the complex conservative structures (*abh-tet*(X4)-IS*CR2-virD2-floR*). The primary difference between the two types, II-1 and II-2, depends on the presence of IS*1R* in the upstream region. Group III (*abh-tet*(X4)-IS*CR2-yheS-cat-zitR*-ΔIS*CR2-virD2-floR*) is the most sophisticated genetic environment in this study, and has a size of 11 kb. Of particular concern is the diversity of *tet*(X4) sources [38, 39, 57], which exacerbates the risk of its spread to different hosts and should attract our attention. Furthermore, the region carrying *tet*(X) in strains of nose swab origin was versatile compared with *tet*(X)-positive strains from other sources (Fig. S8). Further, most strains harboured the genetic environment of group III, so this genetic environment might be advantageous for spreading the *tet*(X4) gene in the pig farm environment and should attract due attention. More interestingly, two types of *tet*(X4) repeat regions (*abh-tet*(X4)-IS*CR2* and *abh-tet*(X4)-IS*CR2-yheS-cat-zitR*-ΔIS*CR2*) were also discovered in this study. Several published reports also found the phenomenon of tandem repeat regions harbouring *tet*(X) [24, 38, 58], but the MICs of tigecycline in strains harbouring different *tet*(X4) copy numbers showed no significant changes, hence the underlying mechanism remains to be investigated.

### Analysis of diverse plasmids carried *tet*(X4)

To identify *tet*(X4) location and further illustrate the associated plasmid contexts, 10 of the plasmids were picked and sequenced by Nanopore sequencing to obtain their complete maps (Table S6). Subsequently, a circular comparison figure was constructed and showed that all the contigs carrying *tet*(X4) were mapped to the plasmid reference pNT1N31-tetX4 (Fig. S9). The comparison results demonstrated that all *tet*(X4) in this study was located on plasmids, implying that plasmids may be the key vector in the horizontal spread of *tet*(X4). It is noteworthy that all the *tet*(X)-carrying plasmids contained IncX1-IncFIA-IncFIB plasmid replicons and showed high similarity with each other. Plasmids carrying the *tet*(X4) gene with the same plasmid replicon type were isolated from different sources in the pig farm, indicating that plasmids carrying *tet*(X4) could transfer between different hosts, which intensifies the risk of *tet*(X4) transmission.

Other plasmids carried the same Inc type as the *tet*(X4)-positive plasmid in this study were also found in the NCBI nr database, mainly collected from cow (p54-tetX) and pig (pG3X16-2-3, pRF10-1_119k_tetX, pRW8-1_122k_tetX, pYPE12-101k-tetX4). However, such *tet*(X4)-positive plasmids of this replicon type have not yet been identified in human sources and most of them were found in pig farms, indicating that pig farms are important reservoirs for such multi-plasmid replicon plasmids and should attract our attention. Further, IncFIA-IncFIB-IncX1 type *tet*(X4)-positive plasmids were only found in *E. coli* [24, 25, 59], and the reasons for this phenomenon deserve further investigation. The phylogenetic tree showed that the IncFIA-IncFIB-IncX1 type plasmid contains not only the *tet*(X4) gene but also *floR*, *sul3*, *tet*(A) and *tet*(M) genes (Fig. S10). The same Inc type plasmids carrying multiple resistance genes were found in various environmental samples, indicating that the different sources contained multiple resistance genes, including that the *tet*(X4) gene in the pig farm environment may be mediated by plasmid transfer.

Tigecycline is a third-generation tetracycline therapeutic used in human medicine and was first applied in clinical therapy in 2005 [60]. Due to its broad-spectrum antimicrobial activity against MDR pathogens, tigecycline is widely used in clinical treatment, leading to the emergence of tigecycline-resistant bacteria. The most common tigecycline resistance mechanism is overexpression of non-specific active efflux pumps or mutations within the drug-binding site in the ribosome [8], and *tet*(X4) gene-mediated tigecycline-resistant strains are rarely reported in clinical specimens [7, 59, 61]. Although tigecycline has not been used in veterinary clinics, an alarming number of *tet*(X4)-carrying *E. coli* isolates have been identified in animals and the environment [7]. This phenomenon may be due to the drug pressure of tetracyclines and other antimicrobials used in veterinary settings.

The current study provides a systematic analysis of *tet*(X4)-positive *E. coli* in the pig farm environment, expanding our comprehensive understanding of the diversity and complexity of *tet*(X4)-bearing plasmids. All detected *tet*(X4)-carrying strains in this study were *E. coli *. Further, *tet*(X4)-positive *E. coli* strains were also detected in several previous studies [9, 24], which implies that *E. coli* is an important carrier for the spread of *tet*(X4). Further, the positive rate for *tet*(X4) strains in this study was higher than that previously reported. This phenomenon is a reminder for us to pay more attention to the rational use of antibiotics in the breeding process. The phylogenetic tree for *E. coli* collected from different sources in the pig farm and the diversity of MLSTs imply that horizontal gene transfer plays an important role in the transmission of *tet*(X4) among *E. coli* strains. We also found an abundance of prophages in bacteria and found a variety of resistance genes and virulence genes in the prophage regions, which deserves further attention. Furthermore, it has been shown that *tet*(X4) usually has a relatively diverse genetic environment in *E. coli* [39], and a similar phenomenon was also found in this study, with it greatly expanding its host range during evolution. The IncX1-IncFIA-IncFIB plasmid carrying the *tet*(X4) gene could form fusion plasmids with different plasmids (IncFII and IncI1), which significantly increased the risk of multi-drug resistance transmission. However, the mechanism of the formation of multiple copies of the *tet*(X4) gene in the strain and how it affected the host strain deserve further attention. The *tet*(A)-v and *tet*(X4) genes can reduce the sensitivity of strains to tigecycline, but whether they have a synergistic effect and their influence on the growth of strains need to be further studied. Furthermore, the fitness costs of the fusion plasmids need to be studied to infer their evolutionary destiny. In summary, a quite high detection rate for ARGs including *tet*(X4) in pig farm environments was observed, warning us to increase monitoring of ARG dissemination in pig farms.

## Supplementary Data

Supplementary material 1
