# Supplementary material for: Distribution and genomic characterization of tigecycline-resistant tet(X4)-positive Escherichia coli of swine farm origin
Source: Microb Genom. 2021 Oct 25;7(10):000667. doi: 10.1099/mgen.0.000667 (PMC8627205; doi:10.1099/mgen.0.000667)
Supplement: Supplementary material 1 [file mgen-7-0667-s001.pdf]

Supplementary Data

Supplementary Table 1. Numbers of *tet*(X4) positive strains and their prevalence among different sources in the swine farm.

Source	Number of samples	Number of *tet*(X4) positive samples	Positive rate[a]	Number of *tet*(X4) positive strains	Number of conjugative strains	Conjugative transfer rate[b]
**All**	**159**	**29**	**18.24%**	**32**	**16**	**50%**
Faeces	36	9	25%	9	4	44.44%
Anal swab	36	11	30.56%	13	3	23.08%
Nose swab	36	5	13.89%	5	5	100%
Soil	21	2	9.52%	2	1	50%
Water	30	2	6.67%	3	3	100%

[a] Positive rate = Number of *tet*(X4) positive samples/Number of samples.
[b] Conjugative transfer rate = Number of conjugable strains/Number of *tet*(X4) positive strains.

Supplementary Table 2. Basic information of the three samples detected with two *tet*(X) positive strains.

Sample IDs	Source	Strain IDs	ST-type	Resistance genes	Replicons
NTW25	water	NT1W25	ST641	*aph(3'')-Ib*, *aph(6)-Id*, *bla*$_{EC\text{-}13}$, *bla*$_{\text{TEM-150}}$, *dfrA5*, *ermB*, *floR*, *mef*(B), *qnrS1*, *sul2*, *sul3*, *tet*(A), *tet*(M), *tet*(X4)	IncFIA(HI1), IncFIB(K), IncFII, IncX1
		NT2W25	ST10	*mph*(A), *bla*$_{\text{EC}}$, *bla*$_{\text{TEM-1B}}$, *dfrA5*, *erm*(B), *floR*, *mef*(B), *qnrS1*, *sul3*, *tet*(A), *tet*(M), *tet*(X4)	IncFIA(HI1), IncFIB(K), IncFII(pHN7A8), IncX1
NTA7	anal swab	NT1A7	ST761	*bla*$_{\text{EC-15}}$, *bla*$_{\text{TEM-1B}}$, *dfrA5*, *floR*, *mef*(B), *qnrS1*, *sul3*, *tet*(A), *tet*(M), *tet*(X4)	IncFIA(HI1), IncFIB(K), IncFII(pCoo), IncX1, IncY
		NT2A7	ST218	*aph(3'')-Ib*, *aph(6)-Id*, *bla*$_{\text{EC-15}}$, *bla*$_{\text{TEM-1B}}$, *dfrA5*, *floR*, *mef*(B), *qnrS1*, *sul3*, *tet*(A), *tet*(M), *tet*(X4)	IncFIA(HI1), IncFIB(K), IncFII, IncX1
NTA19	anal swab	NT1A19	ST716	*aadA5*, *aph(3'')-Ib*, *aph(6)-Id*, *bla*$_{\text{EC}}$, *bla*$_{\text{TEM-1B}}$, *dfrA17*, *dfrA5*, *erm*(B), *floR*, *mef*(B), *mph*(A), *sul1*, *sul3*, *tet*(A), *tet*(M), *tet*(X4)	IncFIA(HI1), IncFIB(K), IncFIB(pHCM2), IncR, IncX1, IncY
		NT2A19	ST716	*aadA5*, *aph(3'')-Ib*, *aph(6)-Id*, *bla*$_{\text{EC}}$, *bla*$_{\text{TEM-1B}}$, *dfrA17*, *dfrA5*, *erm*(B), *floR*, *mef*(B), *mph*(A), *sul1*, *sul3*, *tet*(A), *tet*(M), *tet*(X4)	IncFIA(HI1), IncFIB(K), IncFIB(pHCM2), IncR, IncX1, IncY

Supplementary Table 3. Antimicrobial susceptibility profiles of the *tet*(X4)-positive strains and corresponding transconjugants to different antimicrobials (mg/L).

Isolates	Source	*tet*(X) genetic contexts	Antimicrobials[a]														
			TIG	TET	FFC	DOX	ATM	CQM	KAN	ENR	AMX	OXY	CL	TMP	STR	MIN	MEM
**NT2W25**	**Water**	**III**	64	>64	>64	64	≤0.125	≤0.25	8	>16	>128	>64	≤0.125	>64	16	>128	≤0.03
**NTJ2W25**	**transconjugant**	**III**	16	>64	>64	32	≤0.125	≤0.25	2	≤0.03	16	>64	≤0.125	>64	8	64	≤0.03
**NT1N31**	**Nose swab**	**I-2**	64	>64	>64	128	≤0.125	≤0.25	16	2	>128	>64	≤0.125	>64	16	>128	≤0.03
**NTC1N31**	**transconjugant**	**I-2**	64	>64	>64	64	≤0.125	≤0.25	4	2	>128	>64	≤0.125	>64	8	>128	≤0.03
**NT1N4**	**Nose swab**	**II-2**	64	>64	>64	64	≤0.125	≤0.25	16	1	>128	>64	≤0.125	>64	16	>128	≤0.03
**NTC1N4**	**transconjugant**	**II-2**	32	>64	>64	64	≤0.125	≤0.25	4	1	>128	>64	≤0.125	>64	4	>128	≤0.03
**NT1A25**	**Anal swab**	**III**	32	>64	>64	64	≤0.125	≤0.25	8	2	>128	>64	≤0.125	>64	64	>128	≤0.03
**NTC1A25**	**transconjugant**	**III**	4	>64	>64	32	≤0.125	≤0.25	4	0.5	>128	>64	≤0.125	64	64	>128	≤0.03
**NT1A31**	**Anal swab**	**III**	32	>64	>64	64	0.25	≤0.25	8	0.5	>128	>64	≤0.125	>64	8	>128	≤0.03
**NTC1A31**	**transconjugant**	**III**	32	>64	>64	128	≤0.125	≤0.25	4	4	>128	>64	≤0.125	>64	8	128	≤0.03
**NT1W22**	**Water**	**III**	8	>64	>64	64	≤0.125	≤0.25	8	1	>128	>64	≤0.125	>64	16	>128	≤0.03
**NTC1W22**	**transconjugant**	**III**	16	>64	>64	64	≤0.125	≤0.25	4	16	>128	>64	≤0.125	>64	8	>128	≤0.03
**NT1W25**	**Water**	**III**	64	>64	>64	64	0.25	≤0.25	16	>16	>128	>64	≤0.125	>64	64	>128	≤0.03
**NTC1W25**	**transconjugant**	**III**	32	>64	>64	64	≤0.125	≤0.25	4	4	>128	>64	≤0.125	>64	128	>128	≤0.03
**NT1C1**	**Soil**	**II-1**	32	>64	>64	64	≤0.125	≤0.25	8	0.25	>128	>64	≤0.125	>64	8	>128	≤0.03
**NTJ1C1**	**transconjugant**	**II-1**	16	>64	>64	64	≤0.125	≤0.25	8	≤0.03	16	>64	≤0.125	>64	8	>128	≤0.03
**NT1F25**	**Faeces**	**III**	4	>64	>64	64	≤0.125	≤0.25	8	2	>128	>64	≤0.125	>64	32	>128	≤0.03
**NTJ1F25**	**transconjugant**	**III**	32	>64	>64	32	≤0.125	≤0.25	8	2	>128	>64	≤0.125	64	4	128	≤0.03
**NT1N25**	**Nose swab**	**III**	32	>64	>64	64	64	>128	16	>16	>128	>64	≤0.125	>64	8	>128	≤0.03
**NTJ1N25**	**transconjugant**	**III**	16	>64	>64	64	32	>128	4	1	>128	>64	≤0.125	>64	8	64	≤0.03
**NT1N34**	**Nose swab**	**I-2**	128	>64	>64	64	≤0.125	≤0.25	16	2	>128	>64	≤0.125	>64	16	>128	≤0.03
**NTJ1N34**	**transconjugant**	**I-2**	64	>64	>64	64	≤0.125	≤0.25	8	2	>128	>64	≤0.125	>64	8	>128	≤0.03
**NT1F31**	**Faeces**	**III**	32	>64	>64	64	≤0.125	≤0.25	8	8	>128	>64	≤0.125	>64	8	>128	≤0.03
**NTC1F31**	**transconjugant**	**III**	32	>64	>64	32	≤0.125	≤0.25	4	4	>128	>64	≤0.125	>64	8	>128	≤0.03
**NT1F34**	**Faeces**	**III**	32	>64	>64	128	≤0.125	≤0.25	8	0.5	>128	>64	≤0.125	>64	8	>128	≤0.03
**NTC1F34**	**transconjugant**	**III**	16	>64	8	32	≤0.125	≤0.25	2	4	>128	>64	≤0.125	>64	4	64	≤0.03
**NT1N28**	**Nose swab**	**III**	64	>64	>64	128	≤0.125	≤0.25	16	0.5	>128	>64	≤0.125	>64	16	>128	≤0.03
**NTJ1N28**	**transconjugant**	**III**	32	>64	>64	64	≤0.125	≤0.25	4	4	>128	>64	≤0.125	>64	4	>128	≤0.03

**NT1F10**	**Faeces**	**II-2**	32	>64	>64	128	≤0.125	≤0.25	8	0.5	>128	>64	≤0.125	>64	16	>128	≤0.03
**NTJ1F10**	**transconjugant**	**II-2**	32	>64	>64	64	≤0.125	≤0.25	4	1	16	>64	≤0.125	>64	8	>128	≤0.03
**NT2A7**	**Anal swab**	**III**	16	>64	>64	64	≤0.125	≤0.25	8	2	>128	>64	≤0.125	>64	128	>128	≤0.03
**NTJ2A7**	**transconjugant**	**III**	2	>64	>64	32	≤0.125	≤0.25	2	≤0.03	32	>64	≤0.125	64	4	8	≤0.03
**NT1C4**	**Soil**	**III**	64	>64	>64	64	≤0.125	≤0.25	2	<0.03	>128	>64	<0.125	>64	64	128	≤0.03
**NT1A13**	**Anal swab**	**III**	32	>64	>64	64	≤0.125	≤0.25	4	0.125	>128	>64	<0.125	>64	4	64	≤0.03
**NT1A16**	**Anal swab**	**III**	64	>64	>64	64	≤0.125	≤0.25	2	0.5	>128	>64	<0.125	>64	2	64	≤0.03
**NT2A19**	**Anal swab**	**III**	8	>64	>64	128	≤0.125	0.5	4	4	>128	>64	<0.125	>64	64	32	≤0.03
**NT1A19**	**Anal swab**	**III**	64	>64	>64	64	≤0.125	≤0.25	4	<0.03	>128	>64	<0.125	>64	64	64	≤0.03
**NT1A22**	**Anal swab**	**III**	32	>64	>64	64	≤0.125	≤0.25	4	0.125	>128	>64	<0.125	>64	2	64	≤0.03
**NT1F19**	**Faeces**	**III**	32	>64	>64	64	≤0.125	≤0.25	8	0.25	>128	>64	<0.125	>64	64	64	≤0.03
**NT1F16**	**Faeces**	**III**	16	>64	>64	64	≤0.125	≤0.25	4	0.25	>128	>64	<0.125	>64	16	128	≤0.03
**NT1F13**	**Faeces**	**III**	64	>64	>64	64	≤0.125	≤0.25	1	<0.03	>128	>64	0.25	>64	32	128	≤0.03
**NT1A34**	**Anal swab**	**III**	16	>64	>64	64	≤0.125	≤0.25	4	<0.03	>128	>64	<0.125	>64	4	64	≤0.03
**NT1F22**	**Faeces**	**III**	32	>64	>64	64	≤0.125	≤0.25	2	<0.03	>128	>64	0.5	>64	64	128	≤0.03
**NT1F28**	**Faeces**	**III**	32	>64	>64	128	≤0.125	≤0.25	2	0.125	>128	>64	<0.125	>64	8	64	≤0.03
**NT1A7**	**Anal swab**	**III**	16	>64	>64	128	≤0.125	0.5	2	4	>128	>64	0.5	>64	16	32	≤0.03
**NT1A1**	**Anal swab**	**III**	32	>64	>64	64	≤0.125	≤0.25	1	0.125	>128	>64	<0.125	>64	2	64	≤0.03
**NT1A4**	**Anal swab**	**III**	32	>64	>64	64	≤0.125	≤0.25	4	0.25	>128	>64	<0.125	>64	16	64	≤0.03
**NT1A28**	**Anal swab**	**III**	16	>64	>64	32	≤0.125	≤0.25	2	0.25	>128	>64	<0.125	>64	16	128	≤0.03

[a]**TIG**, tigecycline; **TET**, tetracycline; **FFC**, florfenicol; **DOX**, doxycycline; **ATM**, aztreonam; **CQM,** cefquinome; **KAN**, kanamycin; **ENR**, enrofloxacin; **AMX**, amoxicillin; **OXY**, oxytetracycline; **CL**, colistin; **TMP**, trimethoprim; **STR**, streptomycin; **MIN**, minocycline; **MEM**, meropenem.

Supplementary Table 4. Basic information of 32 *tet*(X) positive strains.

Sample IDs	Biosample	Sequencing Technology	Assembly methods	Completion state of assembly[a]	Num_seqs	Sum_len	Isolation source	Collected date	Location
NT1A1	SAMN16120625	Illumina MiSeq	SPAdes	draft	135	4,781,470	anal swab	2018	China: Nantong
NT1A13	SAMN16120628	Illumina MiSeq	SPAdes	draft	127	4,735,833	anal swab	2018	China: Nantong
NT1A16	SAMN16120629	Illumina MiSeq	SPAdes	draft	136	4,911,356	anal swab	2018	China: Nantong
NT1A19	SAMN16120630	Illumina MiSeq	SPAdes	draft	125	4,854,895	anal swab	2018	China: Nantong
NT1A22	SAMN16120631	Illumina MiSeq	SPAdes	draft	129	4,736,031	anal swab	2018	China: Nantong
NT1A25	SAMN16120632	Illumina MiSeq	SPAdes	draft	113	4,741,934	anal swab	2018	China: Nantong
NT1A28	SAMN16120633	Illumina MiSeq	SPAdes	draft	141	4,836,843	anal swab	2018	China: Nantong
NT1A31	SAMN16120634	Illumina MiSeq	SPAdes	draft	129	4,856,968	anal swab	2018	China: Nantong
NT1A34	SAMN16120635	Illumina MiSeq	SPAdes	draft	167	4,588,704	anal swab	2018	China: Nantong
NT1A4	SAMN16120626	Illumina MiSeq	SPAdes	draft	133	4,734,966	anal swab	2018	China: Nantong
NT1A7	SAMN16120627	Illumina MiSeq	SPAdes	draft	135	4,945,567	anal swab	2018	China: Nantong
NT1C1	SAMN16120636	Illumina MiSeq	SPAdes	draft	131	4,765,887	soil	2018	China: Nantong
NT1C4	SAMN16120637	Illumina MiSeq	SPAdes	draft	124	4,851,737	soil	2018	China: Nantong
NT1F10	SAMN16120638	Illumina MiSeq; Oxford Nanopore MinION	SPAdes; Flye; Unicycler	complete	4	5,001,593	faeces	2018	China: Nantong
NT1F13	SAMN16120639	Illumina MiSeq	SPAdes	draft	125	4,853,670	faeces	2018	China: Nantong
NT1F16	SAMN16120640	Illumina MiSeq	SPAdes	draft	161	4,577,240	faeces	2018	China: Nantong
NT1F19	SAMN16120641	Illumina MiSeq	SPAdes	draft	158	4,576,330	faeces	2018	China: Nantong
NT1F22	SAMN16120642	Illumina MiSeq	SPAdes	draft	127	4,683,777	faeces	2018	China: Nantong
NT1F25	SAMN16120643	Illumina MiSeq; Oxford Nanopore MinION	SPAdes; Flye; Unicycler	incomplete	2	4,739,619	faeces	2018	China: Nantong
NT1F28	SAMN16120644	Illumina MiSeq	SPAdes	draft	136	4,736,267	faeces	2018	China: Nantong
NT1F31	SAMN12996975	Illumina MiSeq; Oxford Nanopore MinION	SPAdes; Flye; Unicycler	complete	4	5,034,814	faeces	2018	China: Nantong
NT1F34	SAMN16120645	Illumina MiSeq; Oxford Nanopore MinION	SPAdes; Flye; Unicycler	incomplete	3	4,896,982	faeces	2018	China: Nantong
NT1N25	SAMN16120647	Illumina MiSeq; Oxford Nanopore MinION	SPAdes; Flye; Unicycler	complete	3	4,871,057	nose swab	2018	China: Nantong
NT1N28	SAMN16120648	Illumina MiSeq; Oxford Nanopore MinION	SPAdes; Flye; Unicycler	incomplete	3	4,914,062	nose swab	2018	China: Nantong
NT1N31	SAMN16120649	Illumina MiSeq; Oxford Nanopore MinION	SPAdes; Flye; Unicycler	complete	3	4,779,075	nose swab	2018	China: Nantong
NT1N34	SAMN16120650	Illumina MiSeq;	SPAdes; Flye;	complete	3	4,777,969	nose swab	2018	China: Nantong

		Oxford Nanopore MinION	Unicycler						
NT1N4	SAMN16120646	Illumina MiSeq	SPAdes	draft	139	4,856,729	nose swab	2018	China: Nantong
NT1W22	SAMN16120651	Illumina MiSeq; Oxford Nanopore MinION	SPAdes; Flye; Unicycler	complete	3	4,862,371	water	2018	China: Nantong
NT1W25	SAMN16120652	Illumina MiSeq; Oxford Nanopore MinION	SPAdes; Flye; Unicycler	complete	4	4,873,400	water	2018	China: Nantong
NT2A19	SAMN16120654	Illumina MiSeq	SPAdes	draft	130	4,851,443	anal swab	2018	China: Nantong
NT2A7	SAMN16120653	Illumina MiSeq	SPAdes	draft	101	5,047,123	anal swab	2018	China: Nantong
NT2W25	SAMN16120655	Illumina MiSeq	SPAdes	draft	133	4,703,970	water	2018	China: Nantong

[a] The incomplete sequences are caused by multiple copies of *tet*(X4).

Supplementary Table 5. Chromosomally mediated CRISPRs spacers specific to plasmids and phages.

Spacer IDs	Matching products	Accession no.	Product code[a]
3	pLD91-1-76kb	NZ_CP042584.1	DUF1380 family protein
2	phage Ss-VASD	KR781488	no
12	cp32-quad	NC_015906.1	no
17	phage P1	MH422554	hp
21	pLD91-1-76kb	NZ_CP042584.1	hp
38	pIOMTU792	NZ_LC542972.1	no
39	phage 118970_sal3	KU927493	putative DNA methyltransferase
42	pLec-476cz	NZ_KY320277.1	DNA (cytosine-5-)-methyltransferase
45	2012C-4221 plasmid unnamed1	NZ_CP027438.1	hp
48	pN069_3	NZ_AP019820.1	hp
50	phage Edno5	MH898687	hp
55	phage ST974-OXA48phi18.2	MK448237	terminase small subunit
66	phage LV-2017	KY271401	portal protein
92	phage S-CAM3	KU686199	hp

[a] no, not detected; hp, hypothetical protein.

Supplementary Table 6. Basic information of ten *tet*(X4)-positive plasmids sequenced in this study.

Plasmid	Isolation source	Plasmid size	Sequencing Technology	Replicons	Resistance genes
pNT1F10-tetX4	faeces	97,500 bp	Oxford Nanopore MinION, Illumina	IncFIA,IncFIB,IncX1	bla_{TEM-1B}, *dfrA5*, *floR*, *mef*(B), *qnrS1*, *sul3*, *tet*(A), *tet*(M), *tet*(X4)
pNT1F25-tetX4	faeces	122,563 bp	Oxford Nanopore MinION, Illumina	IncFIA,IncFIB,IncX1	bla_{TEM-1B}, *dfrA5*, *floR*, *mef*(B), *qnrS1*, *sul3*, *tet*(A), *tet*(M), *tet*(X4)
pNT1F31-tetX4	faeces	115,253 bp	Oxford Nanopore MinION, Illumina	IncFIA,IncFIB,IncX1	bla_{TEM-1B}, *dfrA5*, *floR*, *mef*(B), *qnrS1*, *sul3*, *tet*(A), *tet*(M), *tet*(X4)
pNT1F34-tetX4	faeces	128,941 bp	Oxford Nanopore MinION, Illumina	IncFIA,IncFIB,IncX1	bla_{TEM-1B}, *dfrA5*, *floR*, *mef*(B), *qnrS1*, *sul3*, *tet*(A), *tet*(M), *tet*(X4)
pNT1N25-tetX4	nose swab	147,983 bp	Oxford Nanopore MinION, Illumina	IncFIA,IncFIB,IncX1	bla_{TEM-1B}, *dfrA5*, *floR*, *mef*(B), *qnrS1*, *sul3*, *tet*(A), *tet*(M), *tet*(X4)
pNT1N28-tetX4	nose swab	148,421 bp	Oxford Nanopore MinION, Illumina	IncFIA,IncFIB,IncX1	bla_{TEM-1B}, *dfrA5*, *floR*, *mef*(B), *qnrS1*, *sul3*, *tet*(A), *tet*(M), *tet*(X4)
pNT1N31-tetX4	nose swab	103,246 bp	Oxford Nanopore MinION, Illumina	IncFIA,IncFIB,IncX1	bla_{TEM-1B}, *dfrA5*, *floR*, *mef*(B), *qnrS1*, *sul3*, *tet*(A), *tet*(M), *tet*(X4)
pNT1N34-tetX4	nose swab	103,081 bp	Oxford Nanopore MinION, Illumina	IncFIA,IncFIB,IncX1	bla_{TEM-1B}, *dfrA5*, *floR*, *mef*(B), *qnrS1*, *sul3*, *tet*(A), *tet*(M), *tet*(X4)
pNT1W22-tetX4	water	101,476 bp	Oxford Nanopore MinION, Illumina	IncFIA,IncFIB,IncX1	bla_{TEM-1B}, *dfrA5*, *floR*, *mef*(B), *qnrS1*, *sul3*, *tet*(A), *tet*(M), *tet*(X4)
pNT1W25-tetX4	water	102,128 bp	Oxford Nanopore MinION, Illumina	IncFIA,IncFIB,IncX1	$bla_{TEM-150}$, *dfrA5*, *floR*, *mef*(B), *qnrS1*, *sul3*, *tet*(A), *tet*(M), *tet*(X4)

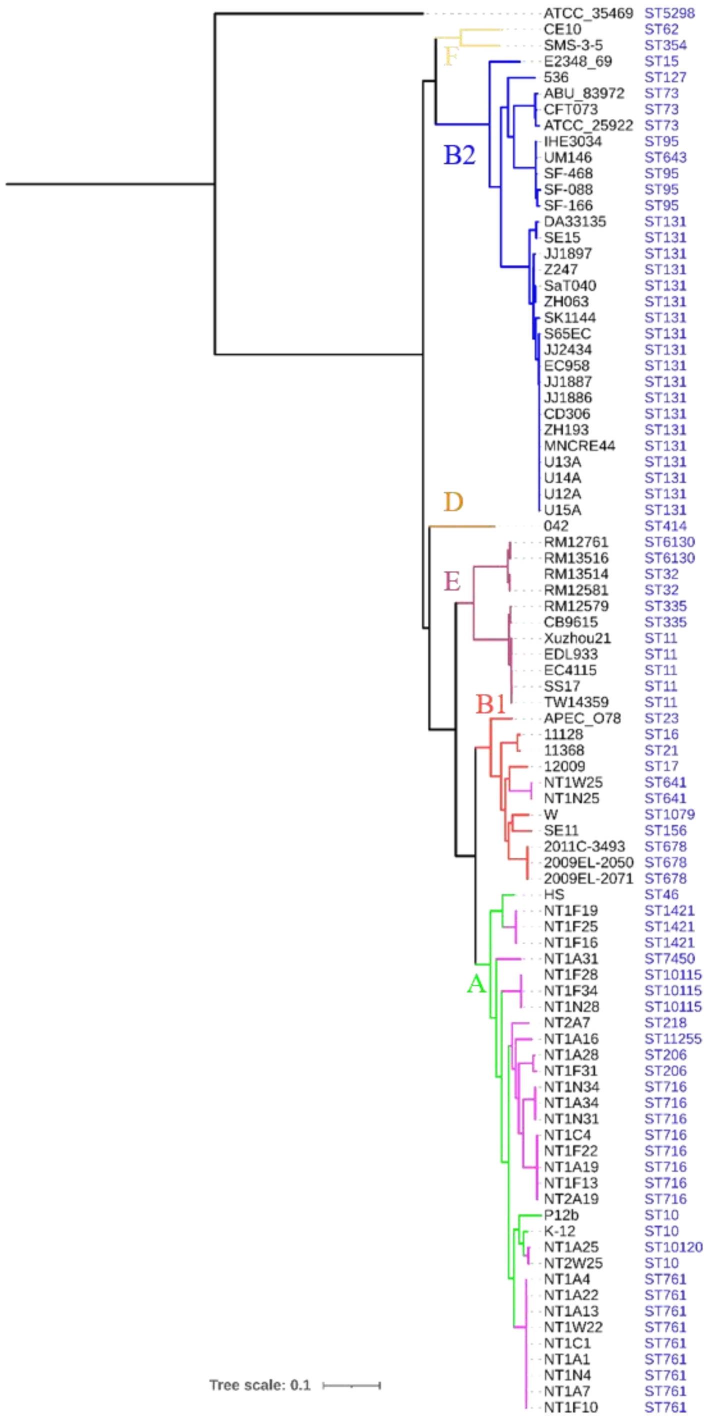

Figure S1. Core-genome phylogeny of 88 *Escherichia coli* strains. Maximum likelihood analysis built from 140,071 core-genome single-nucleotide polymorphisms (SNPs) relative to the reference chromosome NT1F31 (GenBank: CP045190). SNPs were identified with Parsnp v1.2 and represent a 1,622,661 bp core-genome. Phylogeny is rooted according to the actual root by *Escherichia fergusonii* ATCC 35469 (GenBank: CU928158). Branch colours indicate the six main phylogenetic groups where the strains highlighted in magenta are from this study. Branch lengths and scale bar represent number of nucleotide substitutions per site.

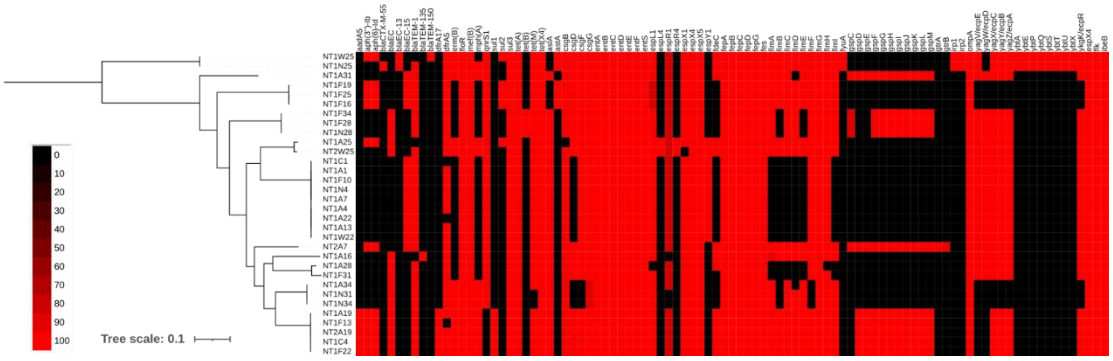

Figure S2. Phylogenetic tree of 32 *tet*(X4)-positive *E. coli* strains. The right part represents the heatmap of all antimicrobial resistance genes and virulence factors in *tet*(X4)-positive isolates collected from the pig farm.

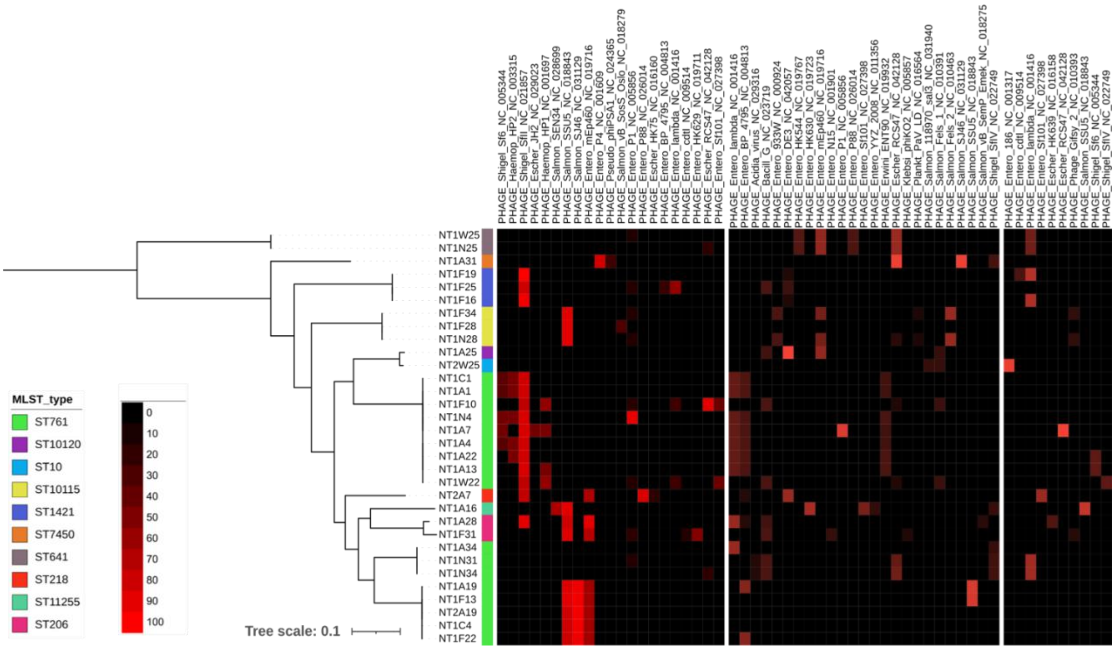

Figure S3. Prophage distribution among 32 *tet*(X4)-positive *E. coli*. The redder the color, the higher the similarity.

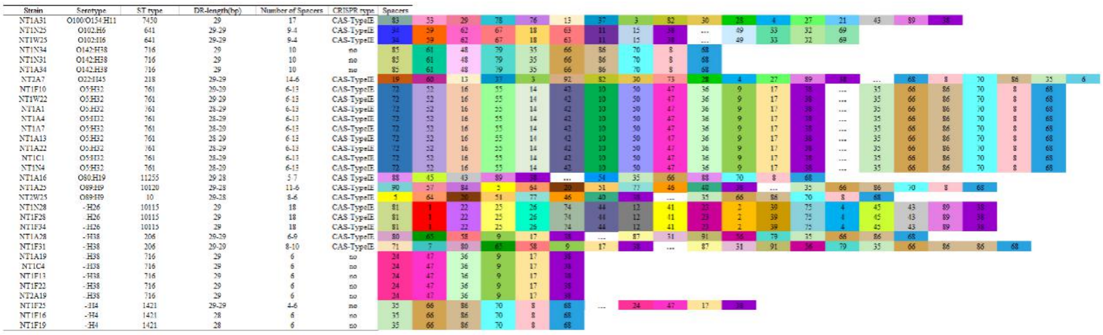

Figure S4. CRISPR allele configuration of 32 *E. coli* strains. The number in the rectangle represents the serial number of the spacer. Replace discrete spacers with "...", and repeat spacers with the same color.

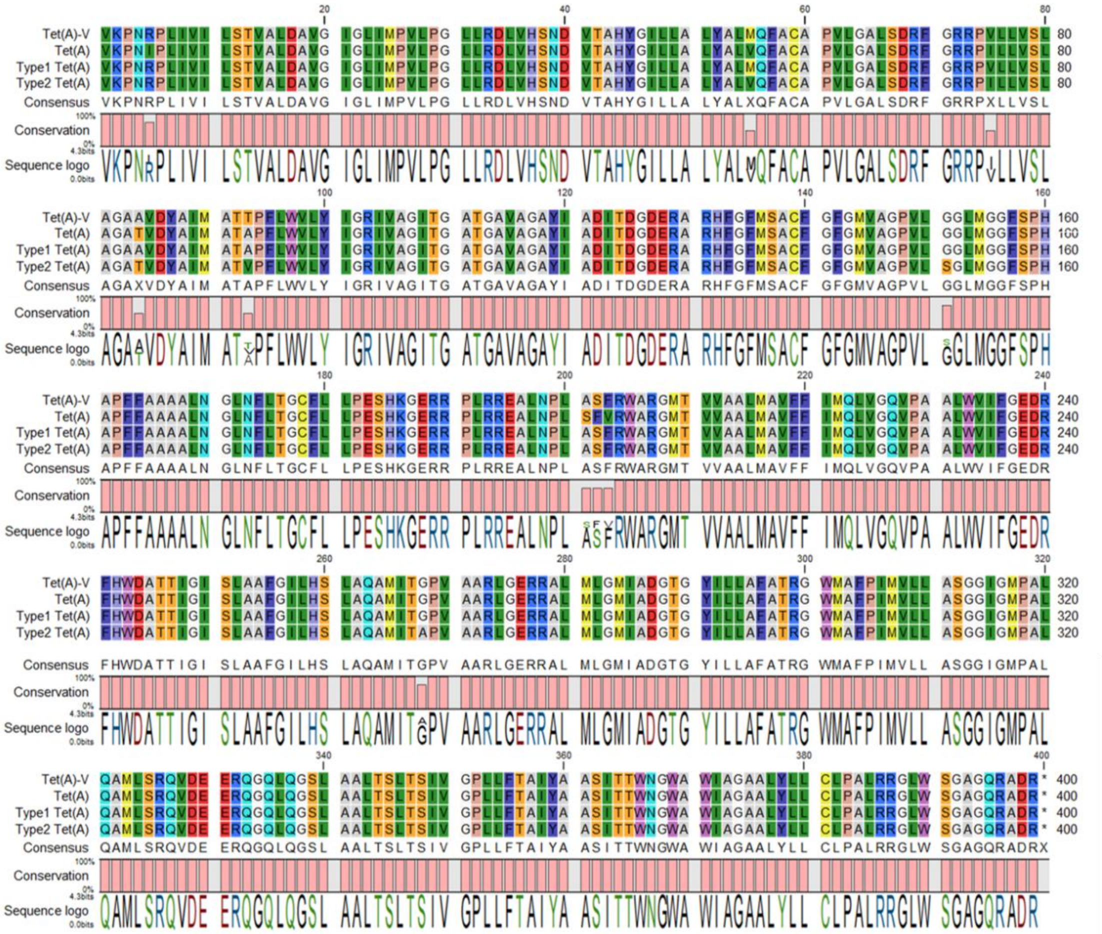

Figure S5. Amino acid sequence alignment of different *tet*(A) variants. Colors in the figure represent different amino acids. Amino acid sequence changed in Tet(A)-V compared with Tet(A) with the following sites: Thr5Arg, Val55Met, IIe75Val, Thr84Ala, Ala93Thr, Ser201Ala, Phe202Ser and Val203Phe.

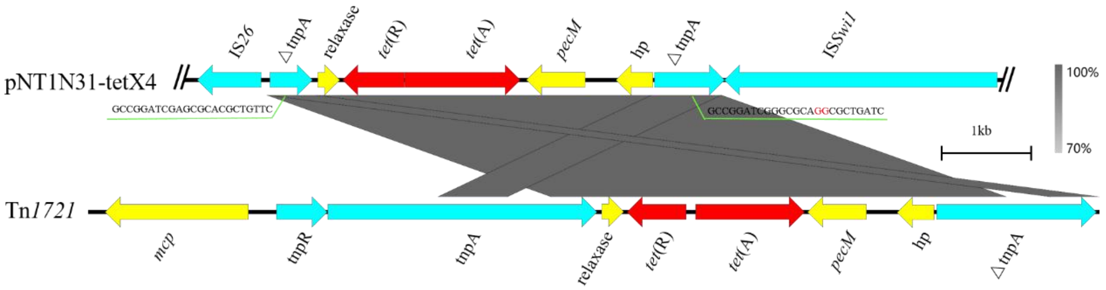

Figure S6. Linear comparison of *tet*(A)-v genetic environment and Tn*1721*. The red arrows and the blue arrows represent the resistance genes and mobile elements, respectively. The gray regions indicate the homologous region between plasmid regions.

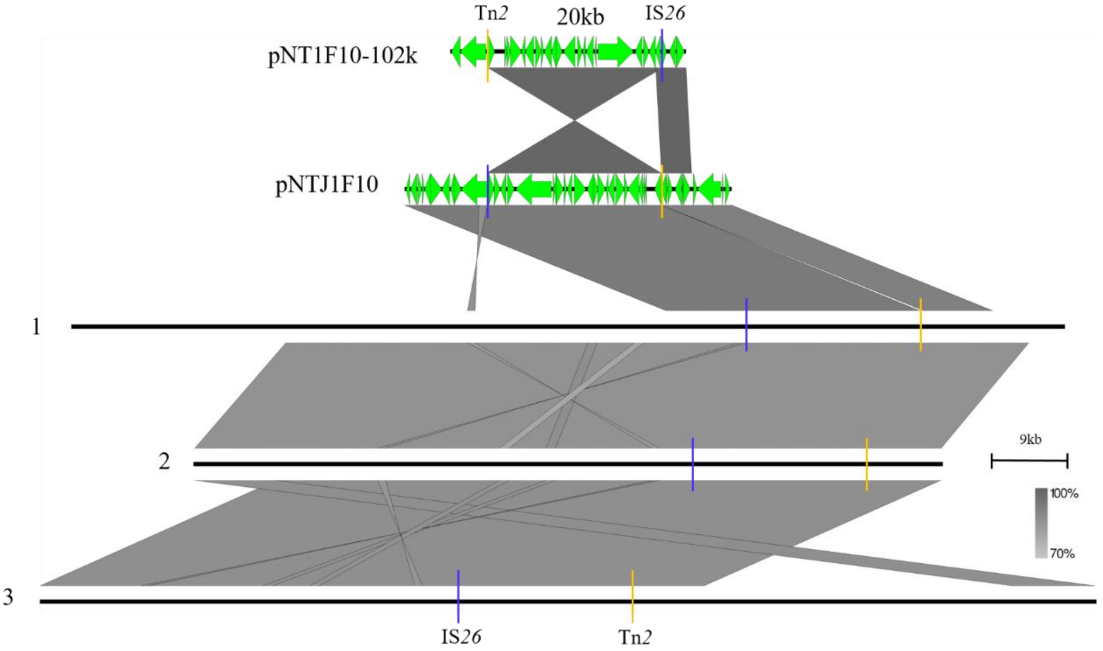

Figure S7. Linear comparison of 20kb region in pNT1F10-102k, pNTJ1F10 and Nanopore sequencing long reads. The gray regions indicate the homologous region between plasmid regions.

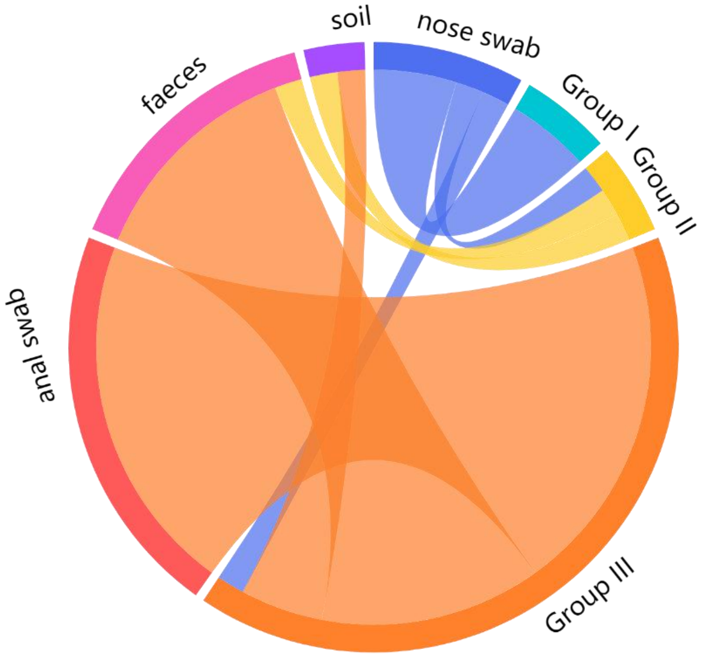

Figure S8. Chord diagram combining the *tet*(X4)-positve strain source and *tet*(X4) genetic environments. A total of 3 groups *tet*(X4) genetic environments and 3 groups strain source.

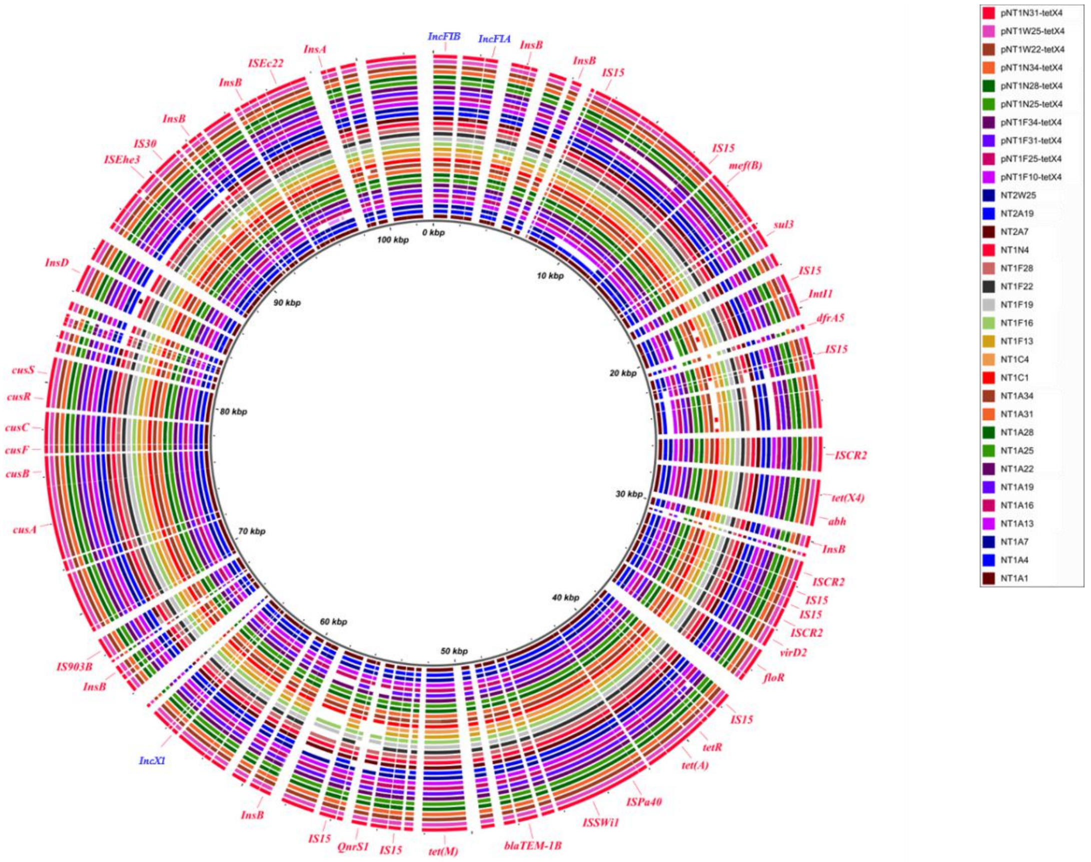

Figure S9. Circular comparison of *tet*(X4)-bearing plasmids. All the plasmids carried *tet*(X4) gene contain the IncX1, IncFIA and IncFIB plasmid replicon. Blank region represented the non-coding sequences.

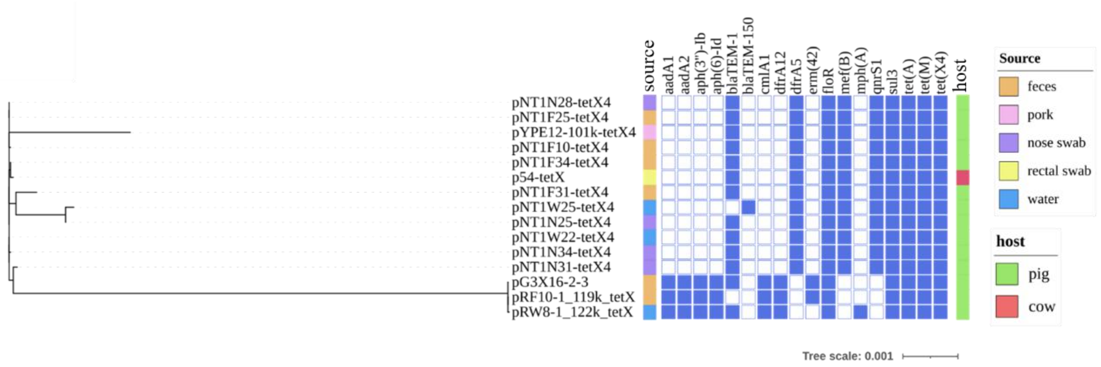

Figure S10. Phylogenetic tree of 10 *tet*(X4)-positive plasmids in this study and other same Inc type plasmids in NCBI. The genes *floR*, *sul3*, *tet*(A) and *tet*(M) were found in all *tet*(X4)-positive plasmids.
